# Pediatric Sepsis Guidelines: Summary for resource-limited countries

**DOI:** 10.4103/0972-5229.63029

**Published:** 2010

**Authors:** Praveen Khilnani, Sunit Singhi, Rakesh Lodha, Indumathi Santhanam, Anil Sachdev, Krishan Chugh, M. Jaishree, Suchitra Ranjit, Bala Ramachandran, Uma Ali, Soonu Udani, Rajiv Uttam, Satish Deopujari

**Affiliations:** **From:** IAP (Intensive Care Chapter), B42 Panchsheel enclave New Delhi 110017, India

**Keywords:** Pediatric, sepsis, septic shock

## Abstract

**Justification:**

Pediatric sepsis is a commonly encountered global issue. Existing guidelines for sepsis seem to be applicable to the developed countries, and only few articles are published regarding application of these guidelines in the developing countries, especially in resource-limited countries such as India and Africa.

**Process:**

An expert representative panel drawn from all over India, under aegis of Intensive Care Chapter of Indian Academy of Pediatrics (IAP) met to discuss and draw guidelines for clinical practice and feasibility of delivery of care in the early hours in pediatric patient with sepsis, keeping in view unique patient population and limited availability of equipment and resources. Discussion included issues such as sepsis definitions, rapid cardiopulmonary assessment, feasibility of early aggressive fluid therapy, inotropic support, corticosteriod therapy, early endotracheal intubation and use of positive end expiratory pressure/mechanical ventilation, initial empirical antibiotic therapy, glycemic control, and role of immunoglobulin, blood, and blood products.

**Objective:**

To achieve a reasonable evidence-based consensus on the basis of published literature and expert opinion to formulating clinical practice guidelines applicable to resource-limited countries such as India.

**Recommendations:**

Pediatric sepsis guidelines are presented in text and flow chart format keeping resource limitations in mind for countries such as India and Africa. Levels of evidence are indicated wherever applicable. It is anticipated that once the guidelines are used and outcomes data evaluated, further modifications will be necessary. It is planned to periodically review and revise these guidelines every 3–5 years as new body of evidence accumulates.

## Introduction

Sepsis is a commonly encountered problem and a major cause of mortality in 80% of children worldwide.[1,2] Till date, published pediatric sepsis guidelines are mostly applicable to developed countries.[3,4] There are no published guidelines for resource-limited countries. A perceived need for simple guidelines particularly applicable to resource-limited countries inspired the Indian Academy of Pediatrics (IAP) Intensive Care Chapter to formulate such guidelines. An expert representative panel appointed by IAP Intensive Care Chapter, met in Delhi on May 31, 2008 to put together evidence-based pediatric sepsis guidelines suitable for resource-limited settings.

## Aims and Objectives

1. To identify levels of resource limitations and feasibility of interventions.
2. To formulate guidelines with reference to consensus sepsis definitions, rapid cardiopulmonary assessment, and management of severe sepsis and shock.

### Available Resource and Limitations

**Figure 1:**
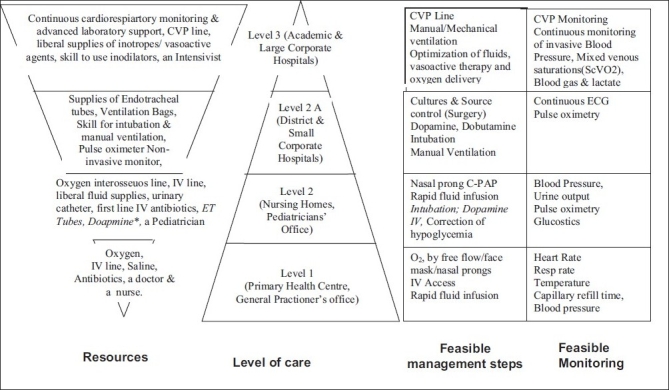
Resources available at different levels of health care facilities in resource-limited countries and feasibility of monitoring and interventions. *Not available universally at all level two facilities

Several resource limitations were identified such as limited availability of pediatric intensive care unit (PICU) beds,[5] in contrast to developed countries,[6] inadequate transport facilities,[7] lack of trained personnel, medications, monitors, infusion pumps, ventilators, and support services such as laboratory, blood bank, and radiology [Figure 1]. In addition, differences in patient population and spectrum of diseases such as malaria and dengue were addressed.[8] Most patients with dengue shock syndrome would respond simply to oxygen and fluid resuscitation, which may not be as aggressive as in septic shock.[9] The fluid management may be different in patients with malaria; one study suggests benefit for the use of albumin.[10] A significant number of children are malnourished who tend to be sicker,[11,12] and there are concerns about the adverse effects of aggressive fluid therapy in these children. The current WHO guidelines on the management of severe malnutrition recommend small fluid boluses and thereafter use of blood transfusion.[13] Finally, rampant misuse of broad spectrum antimicrobials makes it even more challenging to treat sepsis with drug-resistant organisms. Guidelines were developed keeping above-mentioned limitations in mind.

- **Sepsis definitions:** Definitions of sepsis based on International Consensus Conference 2005[14] are presented in Tables 1–3.

**Table 1:** Definitions of sepsis

Systemic Inflammatory Response Syndrome (SIRS) The presence of at least two of the following four criteria, one of which must be abnormal temperature or leukocyte count: Core [oral or rectal] temperature of >38.5 °C or <36°C Tachycardia, in the absence of external stimulus, chronic drugs, or painful stimuli; or otherwise unexplained persistent elevation over a 0.5 h time period or for children <1 year old: bradycardia, in absence of external vagal stimulus, β-blocker drugs, or congenital heart disease; or persistent depression over a 0.5-h time period. Tachypnea for an acute process not related to underlying neuromuscular disease. Leukocyte count elevated or depressed for age [not secondary to chemotherapy-induced leukopenia] or >10% immature neutrophils. Infection A suspected or proven infection caused by any pathogen or a clinical syndrome associated with a high probability of infection. Evidence of infection includes positive findings on clinical examination, imaging, or laboratory tests (e.g., leukocytes in a normally sterile body fluid, perforated viscus, chest radiograph consistent with pneumonia, petechial or purpuric rash, or purpura fulminans) or a positive culture, tissue stain, or polymerase chain reaction test. Sepsis SIRS in the presence of or as a result of suspected or proven infection. Severe Sepsis Sepsis plus one of the following: cardiovascular organ dysfunction OR acute respiratory distress syndrome or two or more other organ dysfunctions. Organ dysfunctions are defined in [Table 3]. Septic Shock In a child with sepsis presence of: Hypotension [systolic BP <70 mmHg in infant; <70 + 2 × age after 1 year of age] or need for vasoactive drug to maintain BP above fifth centile range [dopamine >5 mcg/kg/min or dobutamine, epinephrine, or norepinephrine at any dose] or Signs of hypoperfusion—any three of the following: decreased pulse volume [weak or absent dorsalis pedis pulse], capillary refilling time >3 s, tachycardia heart rate as defined in [Table 2], core [rectal/oral] to peripheral [Skin-toe] temperature gap >3°C, and urine output <1 ml/kg/h [<20 ml/h in >20 kg child] or Sepsis and cardiovascular organ dysfunction as defined in [Table 3]. Multiple Organ Dysfunction The detection of altered organ functions in the acutely ill patient constitutes multiple organ dysfunction syndrome (MODS; two or more organs involvement).

**Table 2:** Age specific upper and/ or lower limits of heart rate to define tachycardia and bradycardia, respiratory rate to define tachypnea, and systolic blood pressure to define hypotensiona

Age group	HR (bpm) Mean (range)	RR (breath/min)	Systolic BP, mmHg (range)	MAP-CVP (mmHg)
Up to 1 months	140 [100–190]	>60	<60	55
2 months to 1 year	130 [80–180]	>50	<70	60
1–5 years	80 [60–140]	>40	<70+ [2 × age in years]	65
6–10 years	80 [60–130]	>30	<70+ [2 × age in years]	65
>10 years	75 [60–100]	>30	<90	65

For heart rate lower values are approximately at 5^th^ percentile and upper values are at 95^th^ percentile for blood pressure, the values are at 5^th^ percentile and for respiratory rate the values are at 5^th^ percentile and for respiratory rate the values are at 95^th^ percentile

**Table 3:** Organ dysfunction criteria

Cardiovascular Dysfunction(a)
Hypotension [systolic BP <70 mmHg in infant; <70 +2 × age after 1 year of age] or
Need for vasoactive drug to maintain BP above fifth centile range [dopamine >5 mcg/kg/min or dobutamine, epinephrine, or norepinephrine at any dose] or
Signs of hypoperfusion–any three of the following: decreased pulse volume [weak or absent dorsalis pedis pulse], capillary refilling time >3 s, tachycardia [heart rate as defined in Table 2], core [rectal/oral] to peripheral [Skin-toe] temperature gap >3°C, and urine output <1 mL/kg/h [<20 mL/h in >20 kg child].
In early stage, there is an increase in heart rate and poor peripheral perfusion in form of weak pulse and prolonged capillary refill time. Hypotension occurs late, and may lead to precipitous cardiac arrest.
Respiratory Dysfunction(b)
Proven need for supplemental oxygen(c) or >50% FIO_2_ to maintain saturation >92% or
Need for nonelective mechanical ventilation(d) or
PaO_2_/FIO_2_ <300 in absence of cyanotic heart disease or pre-existing lung disease or
PaCO_2_ >65 torr or 20 mmHg over baseline PaCO_2_
Neurologic Dysfunction
Glasgow Coma Score <11 or
Acute change in mental status with a decrease in Glasgow Coma Score >3 points from abnormal baseline
Hematologic Dysfunction
Platelet count <80000/mm^3^ or a decline of 50% in platelet count from highest value recorded over the past 3 days [for chronic hematology/oncology patients] or
International normalized ratio >2
Renal Dysfunction
Serum creatinine >1 mg/dL
Hepatic Dysfunction
Total bilirubin >4 mg/dL [not applicable for newborn] or, alanine transaminase 2 × upper limit of normal for age

a See Table 2

b Acute respiratory distress syndrome must include a P_a_O_2_/FIO_2_ ratio <200 mmHg, bilateral infiltrates, acute onset, and no evidence of left heart failure. Acute lung injury is defined identically except the PaO2/FIO2 ratio must be <300 mmHg.

c Proven need assumes oxygen requirement was tested by decreasing flow with subsequent increase in flow if required

d In postoperative patients, this requirement can be met if the patient has developed an acute inflammatory or infectious process in the lungs that prevents him or her from being extubated.
- **Rapid cardiopulmonary assessment and clinical examination:** Assessment should be prompt and comprehensive. During clinical assessment one must note following points:
  1. **Appearance:** Restlessness, agitation, anxiety, progressive lethargy, and decreased responsiveness are signs of impaired mental status.
  2. **Airway patency and stability.**
  3. **Breathing:** Respiratory rate is increased in response to tissue hypoxia and to compensate for metabolic acidosis. Progressive worsening of respiratory distress (tachypnea, nasal flaring, suprasternal, intercostal, and subcostal retractions) with bilateral rales or wheezes or unequal breath sounds on auscultation are signs of primary focus of infection in lungs, or early acute respiratory distress syndrome (ARDS).
  4. **Circulation (Cardiovascular):** Heart rate, adequacy of central and peripheral pulse, systolic and diastolic blood pressure, skin color, capillary refill time (CRT), and temperature of extremities should be noted.
    Tachycardia occurs early in response to falling cardiac output and is the most significant physical findings in septic shock.
    ***Blood pressure***: A fall in blood pressure is a late manifestation of low-cardiac output in children. Children can prevent reduction in blood pressure by vasoconstriction, and an increase in heart rate and may have features of poor peripheral perfusion in presence of normal blood pressure. Diastolic blood pressure falls early causing wide pulse pressure as vascular tone begins to decrease. Systolic blood pressure begins to fall causing narrow pulse pressure once hemodynamic compromise is severe.
    Hepatomegaly and jugular venous distension with gallop rhythm may signify predominant cardiac involvement as part of septic myocardial depression or myocarditis.
    Petechial rash may be present in meningococcemia or disseminated intravascular coagulation.
    ***Capillary refill time (CRT)***: Capillary refill time of more than 3sec is always abnormal. In warm phase of septic shock, CRT may be normal; however, signs of hyperdynamic circulation (bounding pulse, widened pulse pressure, and hyperdynamic apex beat) are present. Warm shock if untreated will progress to cold shock. Cold shock is more common than warm shock. In older children, cold peripheries, poorly felt pulses, and prolonged CRT are harbingers of shock.
  5. **Urine output:** Oliguria is common and may progress to anuria. Assessment of urine output in last 6 hours is helpful.
    In severe cases, patient may present with cardiopulmonary failure or cardiopulmonary arrest; both situations need aggressive hemodynamic support as well as endotracheal intubation and ventilatory support for survival.
    A time-sensitive protocolized approach to resolve shock in severe sepsis should be implemented with an effort to resolve shock in the initial hours of resuscitation as it is associated with steep decline in mortality rate[15–17] (Level 1).
- **Guidelines for Management of Severe Sepsis and Shock**
  For simplicity sake, components of this flow chart are divided into four steps (I-IV) to address recommended interventions according to clinical condition, time, and available resources **[Flow chart: Figure 2a, b]**. Grading of the literature and levels of recommendations is based on American College of Critical Care Medicine (ACCM) criteria [Table 4].

**Table 4:** ACCM guidelines for evidence-based medicine rating system for strength of recommendation and quality of evidence supporting the references

Rating System for References
(a) Randomized, prospective controlled trial.
(b) Nonrandomized, concurrent or historical cohort investigations.
(c) Peer-reviewed, state-of-the-art articles, review articles, editorials, or substantial case series.
(d) Non-peer-reviewed published opinions, such as textbook statements or official organizational publications.
Rating System for Recommendations
Level 1: Convincingly justifiable on scientific evidence alone.
Level 2: Reasonably justifiable by scientific evidence and strongly supported by expert critical care opinion.
Level 3: Adequate scientific evidence is lacking, but widely supported by available data and expert opinion.

**Figure 2a:**
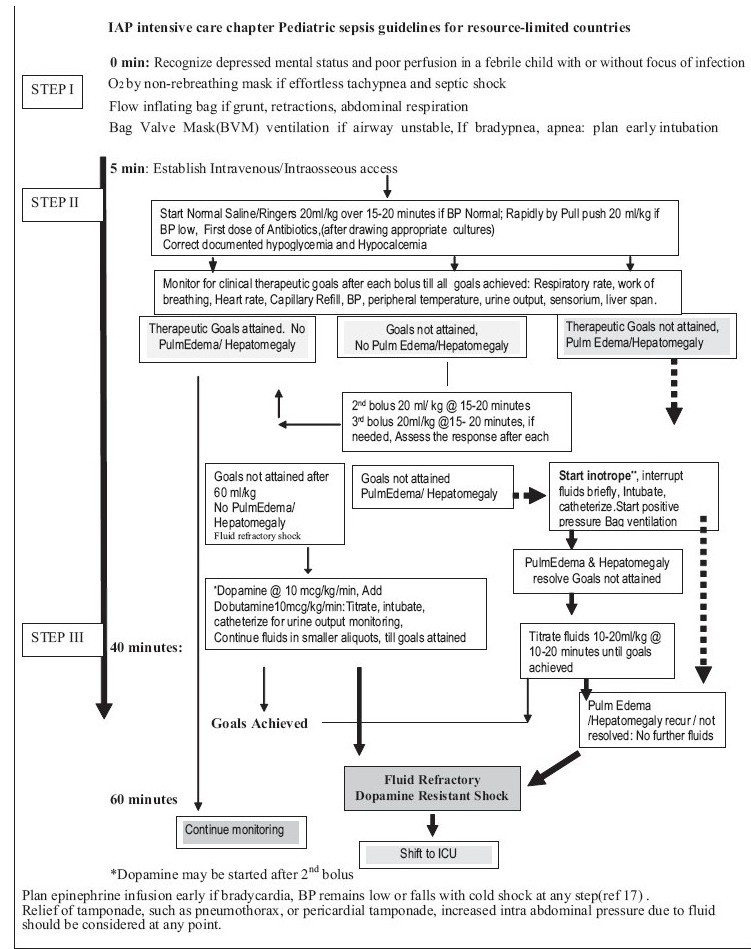
IAP intensive care chapter Pediatric sepsis guidelines for resource limited countries

**Figure 2b:**
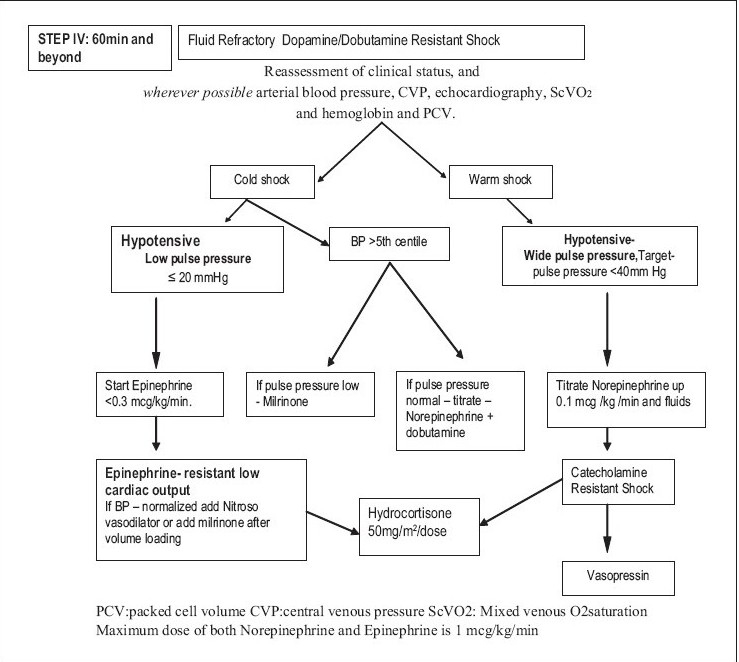
IAP intensive care chapter Pediatric sepsis guidelines for resource limited countries

### STEP 1: 0–5 min:

- Recognize depressed mental status and decreased perfusion by rapid cardiopulmonary assessment.
- Begin high flow oxygen (Level 3).
- Establish intravenous/intraosseous access (Level 2).
- Venturi masks or non-rebreathing mask may be used for high flow oxygen therapy(Level 3).

All of the above are readily achievable in first 5 minutes.

If airway is unstable or the patient is lethargic or unresponsive and adequate oxygenation and ventilation is not achieved, bag-valve mask ventilation should be started and early endotracheal intubation and mechanical ventilation should be planned (level 3). Other indications for intubation are hypotension on arrival or during therapy, convulsive seizures refractory to two doses of benzodiazepine, persistently low Glasgow Coma Scale (GCS) of less than eight and signs of increased intra-cranial pressure. Implementation of this step may take additional time encroaching upon the interventions expected in next 60 min as per the guidelines.

### STEP II: 5–40 min:

- ***Initial fluid resuscitation***: Rapid infusion of 20 mL/kg isotonic saline each, up to 60 mL/kg, titrated toward achievement of therapeutic goals of shock resolution [Table 5] or unless rales or hepatomegaly develop (Level 1).

**Table 5:** Therapeutic endpoints of resuscitation of septic shock

Normalization of the heart rate Capillary refill of <2sec Well felt dorsalis pedis pulses with no differential between peripheral and central pulses Warm extremities Normal range of systolic pressure and pulse pressure Urine output >1ml/kg/hour Return to baseline mental status tone and posture Normal range respiratory rate Other end-points that have been widely used in adults and may logically apply to children include central venous pressure of 8–12 mmHg
- Fluid therapy by peripheral or intraosseous access should be initiated while adequate control of airway, and breathing is being accomplished.
- A second peripheral IV line or central line should be established if feasible (for possible inotrope: Dopamine) (Level 2).
- Antibiotics should be started (third generation cephalosporin and an aminoglycoside) (Level 2).
- Hypoglycemia and hypocalcemia should be started (Level 2).

Volume replacement with 20 mL/kg of isotonic solutions such as normal saline or Ringers lactate can be safely given and repeated if necessary. Typically, 40–60 mL/kg may be required to correct hypovolemia;[18] in some the need may be as high as 120 mL/kg in first hour. It has been suggested that malnourished child may get fluid overloaded with aggressive volume replacement; caution and a slower rate of infusion are advised (Level 3). This issue needs to be systematically studied.

Clinical scenarios where larger volumes are needed to achieve therapeutic end points are warm septic shock and shock due to gastro-intestinal sepsis. Presence of pulmonary edema and shock is an indication that more fluids may be needed to resolve shock.[19] Repeated assessment helps to decide whether further fluids may be given, or stopped and inotrope initiated and intubation and mechanical ventilation may be initiated. It also helps to decide whether further fluids may be titrated after intubation and inotrope infusion.[17,19]

- Choice of fluid for volume replacement
  We recommend that isotonic crystalloid such as Ringers Lactate or Normal saline be used for the initial fluid resuscitation in septic shock (Level 1).[9,18,20,21]
- Method of fluid administration

We suggest that fluids are given in boluses of 20 mL/kg (Level 1); in hypotensive patients as rapidly as possible by pull-push method using a three-way stop-cock (Level 1), and in others by gravity method over 15–20 min should be preferred (Level 2). Infusion pumps are ideal but not always available.

The ACCM guidelines recommend administration of the boluses as fast as possible which can only be administered by pull–push method using a three-way stop-cock.[22] However, a recent prospective study from India shows that administration of fluids by pull–push method using a three-way stop-cock increased the incidence of hepatomegaly /pulmonary edema and a greater need for intubation.[17]

Development of pulmonary edema and hepatomegaly should be anticipated during fluid administration. In some patients, evidence of pulmonary edema and hepatomegaly may be present on arrival, as ARDS and myocardial dysfunction may co-exist in severe sepsis. Clinical signs suggestive of myocardial dysfunction or pulmonary edema on arrival or its development during fluid therapy are shown in [Table 6].

**Table 6:** Sign of pulmonary edema and myocardial dysfunction

Airway: Airway instability, froth, new-onset cough
Breathing: Decreased or increased respiratory rates requiring respiratory support in the absence of neuromuscular diseases, onset of grunt, retractions, abdominal respirations, new rales or wheeze, drop in saturations
Circulation: bradycardia, gallop, hypotension, hepatomegaly
Disability: Agitation, fighting the mask, combativeness and thirst for water
If, any one or a cluster of signs of deterioration are noted during fluid therapy, further fluid administration is discontinued, an appropriate inotrope infusion initiated and intubation is performed.

Other practical ways to assess fluid overload are jugular venous distension, heart size, and pulmonary congestion on chest radiograph (Level 3). Measurement of CVP and bedside echocardiography should be used at tertiary care centers, if available to assess adequacy of intravascular volume, cardiac function, and signs of fluid overload (Level 2).

Patients who develop pulmonary edema and hepatomegaly after fluid boluses should be intubated and given positive pressure ventilation. Care must be taken to provide ventilation with positive end expiratory pressure (PEEP).[19,23] This can be achieved in resource-limited setting using the using self-inflating bag with PEEP valve or Mapleson C-Circuit/Bain's circuit if a mechanical ventilator is not available.

If shock persists following 60 mL/kg fluid and no signs of pulmonary edema/hepatomegaly are noted, elective intubation should be performed. Since shock can worsen during or following intubation, initiation of an appropriate inotrope infusion often improves the safety profile of this procedure, particularly in warm shock.

Achievement of all therapeutic goals [Table 5] is needed to define shock resolution in fluid and inotrope responsive shock. Discontinuing fluid therapy based on achievement of some and not all the goals may result in inadequate resuscitation.

### Early antibiotic therapy and infection control

Antibiotics should be administered within 1hour of the identification of severe sepsis, if possible, after appropriate cultures have been obtained (Level 1). Early antibiotic therapy is as critical for children with severe sepsis as it is for adults.[24]

**Choice of initial antibiotic therapy:** The initial empiric antibiotic therapy should include one or more drugs that have activity against the likely pathogens and that penetrate the presumed source of sepsis [Table 7]. Commonly used antibiotics include a third generation cephalosporin such as ceftriaxone and an aminoglycoside such as amikacin (Level 3).

**Source Control:** Every patient presenting with severe sepsis should be evaluated for the presence of a focus of infection that is amenable to source control measures, e.g., drainage of an abscess, debridement of infected necrotic tissue, removal of a potentially infected device, etc.

### Hypoglycemia

Hypoglycemia should be checked for and corrected (Level 2). Hyperglycemia should be avoided (Level 2).

Hypoglycemia can have devastating neurological consequences and should be diagnosed early and treated immediately[25] (Level 1). Hypoglycemia has been shown to be associated with morbidity and mortality in critically ill children with very severe pneumonia,[26] malaria, and severely ill malnourished children.[27] Hyperglycemia also has been shown to be associated with morbidity and mortality in critically ill similar to the hypoglycemia.[28,29] However, the effects of intensive glucose control on mortality in critically ill children are unknown, and insulin therapy may result in hypoglycemia.[30] One may consider use of insulin only if the child had significant glycosuria and polyuria leading to difficulty in fluid management.

### Calcium and Hypocalcemia

Before cardiac output and perfusion pressure are restored with drugs, ionized hypocalcemia that might impair cardiac performances should be corrected (Level 2).

Ionized hypocalcemia is common in neonates and children with sepsis admitted to PICU.[31,32] Administration of calcium in septic patients with ionized hypocalcemia may transiently improve blood pressure.[33] However, there is no evidence to suggest a survival benefit.[34]

### Monitoring and Therapeutic Endpoints

Meticulous clinical monitoring for therapeutic endpoints without high technology facilities has shown a dramatic reduction in mortality in Vietnamese children presenting with moderate dengue shock syndrome[9] and in Indian children treated for septic shock.[17]

End-points such as O_2_ saturation, and CVP can be monitored at secondary level facilities. Use of cardiac monitor can give reliable continuous heart rate (HR) record. In absence of a monitor, HR could be determined by auscultation periodically; this may be done before, during, and after a fluid bolus has been administered.

**Blood Pressure:** BP monitoring assists to regulate rate of fluid infusion, the need for vasoactive agents and further titration. In vasodilatory or warm shock, with wide pulse pressure narrowing of pulse pressure is an additional therapeutic goal.

### Limitations of Clinical Therapeutic End Points

All the clinical end points may not be applicable in some patients.

While normalization of heart rate is one of the most reliable signs of shock resolution, other causes of tachycardia may be fever, anxiety, pain, and SIRS. It may also be the only sign of ongoing seizure activity in a sedated, muscle-relaxed child. Anti-pyretic and analgesics, anti-seizure medications, source control and mother's close proximity can often help in achievement of normal range of heart rate in appropriate clinical scenarios. On the other hand heart rate, which falls within the normal range for age, in the presence of severe respiratory distress or impending respiratory failure and shock, is an ominous sign (of imminent cardiac arrest).

Poor peripheral perfusion may be the result of cool environmental temperatures in very young infants. Recognition and resolution of shock in these young patients will depend on normalization of mental status, respiratory rates, and heart rates.

There are concerns about the use of capillary refill and pulse volume, as there may be significant interobserver variability.[35]

Accurate urine output monitoring by catheterization in fluid unresponsive shock is useful, especially in settings without access to CVP monitoring.

### Unresolved Issues

1. Time to achieve various therapeutic endpoints may be variable. There are no evidence-based guidelines for defining expected time frame of response for each of the monitoring parameters.
2. Arterial blood gases (ABGs) and lactate estimations are available in a few centers; in others this cannot be used. Use of mixed venous oxygen saturations (ScVO_2_) is still beyond reach of most centers.
3. Ability to place central lines particularly subclavian or internal jugular vein is still limited.
4. In children with shock, the noninvasive BP measurements may be unreliable and invasive intra-arterial BP is ideal; it may not be feasible in majority of resource-limited centers.
5. Echocardiography for determining the cardiac filling is also not practical in many centers.
6. Precise therapeutic end-points for severely malnourished children are unknown.

### STEP III: 40–60 min:

- Recognize Fluid Refractory Shock: Begin inotrope by intravenous or intraosseous (IO) route; Dopamine up to 10 μg/kg/min (Level 2).
- Obtain central venous access and airway if needed and feasible (Level 1).

Following adequate intravascular volume repletion, continued presence of hypotension and/or poor perfusion (fluid refractory shock) warrants the consideration of vasoactive therapy, which should be goal directed.[36,37]

The expert group agrees with the use of dopamine as the first-line vasopressor for fluid refractory hypotensive shock in the setting of low-systemic vascular resistance. Children with septic shock more often have myocardial dysfunction and low-cardiac output. Hence, it is preferable to combine inotropy with a vasopressor effect. Dopamine with or without dobutamine can be used as first-line drugs for giving this kind of support (Level 2). In children, the age-specific insensitivity to dopamine has to be kept in mind before starting dopamine particularly in infants <6 months.[3,38]

### STEP IV: 60 min and Beyond

- Recognize dopamine resistant shock.
- Transfer to PICU.
- If possible, monitor CVP, echocardiography, mean arterial pressure (Level 2).
- Titrate fluids and vasoactive drugs to resolve shock based on CVP, echocardiography to achieve therapeutic goals.
- Reverse cold shock-resistant to dopamine (normal or low blood pressure) titrate central epinephrine (0.05–0.3 μg/kg/min)) (maximum dose 1 microgram/kg/min) (Level 2).

(vi) Reverse warm shock with wide pulse pressure and/or low blood pressure by titrating central norepinephrine (Level 2).

(v) Begin hydrocortisone (50 mg/m^2^/24 h) if child is at risk for absolute adrenal insufficiency (Level 2).

When a child in septic shock does not improve and the goals of treatment are not achieved even after dopamine and or dobutamine infusion, the shock is labeled as *fluid refractory, dopamine/dobutamine-resistant shock*. Dopamine-resistant shock may reverse with epinephrine or norepinephrine infusion [Figure 2b].

Some of pediatric patients may have adult-type manifestation of high cardiac output, vasodilatation, and hypotension. Clinically, it will manifest as tachycardia, flush capillary refill, low-to-low normal blood pressure and wide pulse pressure (warm shock). A vasopressor such as norepinephrine is the drug of choice in such patients. It should be used only to restore adequate values of mean arterial pressure that is sufficient to restore urine output. The usual dose is 0.05–1.00 μg/kg/min.

Children with septic shock more often have myocardial dysfunction with intense compensatory vasoconstriction. This leads to a state of low-cardiac output, with high-cardiac filling pressure and high-systemic vascular resistance, which clinically manifests as tachycardia, signs of hypoperfusion, prolonged capillary refill, cold extremities and low-to-low normal blood pressure and narrow pulse pressure (cold shock). An inotrope such as epinephrine is the drug of choice. The dose range is 0.05–1.00 μg/kg/min.

The low-cardiac output state, characterized by persistent narrow pulse pressure and/or prolonged capillary refill even after use of dopamine may be improved with addition of dobutamine (up to 20 μg/kg/min) or low-dose epinephrine (<0.3 μg/kg/min) (Level 2B).

At various stages of sepsis or the treatment thereof, a child may move from one hemodynamic state to another. Vasopressor or inotrope therapy should be used according to the clinical state.[3]

### Corticosteroids in Septic shock

Corticosteroids should not be used routinely in all children with septic shock. The group recommends stress doses of hydrocortisone 50 mg/m^2^/dose every 6 h until reversal of shock for pediatric sepsis patients with catecholamine-resistant shock and suspected or proven adrenal insufficiency (Level 2).[39,40]

Up to this point most of the interventions can be performed in a peripheral setting to be followed as the guideline in resource-limited situation. Further management requires transfer of the patient to a PICU, reassessment of the patient's clinical status, arterial blood pressure, CVP, echocardiography and hemoglobin and packed cell volume (PCV). Generally, a low CVP will be an indication for more fluids, low blood pressure for more vasopressors, poor contractility of myocardium on echocardiography for titrating the dose of inotropes and low PCV, an indication for packed cell transfusion.

### Further Management and Other Issues

## Vasoactive Drug Therapy: Further Titration

At this stage, children in shock may be classified into two broad categories: warm shock and cold shock.

Children in cold shock may be further categorized in two subgroups. (i) *Children with low BP*. In these children, the dose of epinephrine should be titrated to achieve normal mean arterial pressure for age. Once this is achieved but the other goals of therapy are not yet achieved, one should consider adding a vasodilator such as nitroprusside and nitroglycerine, with very short half-life, or milrinone[41] having both vasodilator as well as inotropic effects. Nitrosovasodilators are used as first-line therapy for children with epinephrine-resistant low-cardiac output and elevated systemic vascular resistance. Use of milrinone (50–75 mg/kg/min) should be strongly considered if low-cardiac output and high-vascular resistance-state persists in spite of epinephrine and nitrosovasodilators. Starting milrinone may require additional fluid bolus, and titrating up the dose of epinephrine to check the vasodilatation and maintain BP.

Second category is that of children with normal BP. In these children, further action would depend on the pulse pressure. If the pulse pressure is low, milrinone would be the drug of choice (Level 1). However, if the pulse pressure is normal or high, norepinephrine and doubtamine should be titrated up.

### Vasopressin in Shock

Vasopressin therapy may be considered as a last resort if patient has warm shock with low blood pressure unresponsive to norepinephrine.[42,43] In pediatric patients, suggested dose is 0.3–2 milliunits/kg/min [equivalent to 0.0003 to 0.002 units/kg/min or 0.01 to 0.12 units/kg/h]. The infusion should be titrated to optimize blood pressure and perfusion.

### Drugs: Practice Points

Accurate dose delivery is an important component of vasoactive drug therapy. This can only be achieved with infusion pumps. When infusion pumps are not available, the infusions may be given using microinfusion sets whose drop size has been standardized. Mixing of more than one vasoactive drug in the same infusion set or infusion syringes is not recommended even when limited numbers of intravenous access ports are available. These drugs can be infused through the intraosseous route till the time that an intravenous access becomes available.

A meticulous search for the causes of persistent catecholamine-resistant shock should be made if therapeutic goals are not achieved in spite of adequate volume loading and high doses of appropriate vasoactive agents. One must rule out mechanical causes of catecholamine-resistant shock such as tamponade because of pericardial effusion, pneumothorax, or increased intrabdominal pressure.

### Blood and Component Therapy

Optimal hemoglobin for a critically ill child with severe sepsis is not known. A Canadian multicenter trial[44] strongly argues in favor of a restrictive transfusion strategy recommending RBC transfusions to only those critically ill children whose Hb is ≤7 g/dL. However, this study excluded children with hemodynamic instability, therefore, the results cannot be extrapolated to children with septic shock.

The adult trial used a goal of 30% PCV (approx. 10 g/dL Hb) during the resuscitation phase of septic shock along with other interventions and showed a clear benefit.[37] Hence, a recommendation for maintaining a somewhat higher Hb level of 10 g/dL during the resuscitation phase is being made here too.

These recommendations may not apply to premature infants, children with severe hypoxemia, or cyanotic heart disease and to children who are actively bleeding.

### Fresh Frozen Plasma

Correction of coagulation abnormalities does not improve outcome in all the patients[45] and unnecessarily exposes the child to the risks of blood product transfusions. Hence, fresh frozen plasma (FFP) is indicated in patients with coagulation abnormality having any of the following: active bleeding, before surgery, before invasive procedure, and to reverse warfarin effect. Routine use of FFP to correct laboratory clotting abnormalities is not indicated. When required, the FFP infusion should be given relatively rapidly to achieve effective factor levels.

### Intravenous Immunoglobulins

Although some pediatric studies have supported the use of intravenous immunoglobulins (IVIG) for severe sepsis,[46,47] large clinical trials and recent consensus guidelines[48] do not recommend the widespread use of IVIG in patients with severe sepsis or septic shock.

### Deep Vein Thrombosis Prophylaxis

Use of deep vein thrombosis (DVT) prophylaxis is recommended in postpubertal children with severe sepsis (Level 2).

#### Stress Ulcer Prophylaxis

Therapy may be individualized. There are no graded recommendations.

#### Renal Replacement Therapy

Continuous veno-venous hemofiltration may be clinically useful in children with anuria/severe oliguria and fluid overload. There are no graded recommendations due to lack of pediatric studies.

## Summary

The recommendations include use of rapid cardiopulmonary assessment and greater use of physical examination for achieving therapeutic endpoints. Early fluid resuscitation (crystalloid or colloids) based on weight with 40-60 mL/kg or higher may be needed.

Early mechanical ventilation should be considered if hemodynamic instability continues beyond fluid therapy. Decreased cardiac output and increased systemic vascular resistance tend to be the most common hemodynamic profile. Dopamine with or without dobutamine is recommended as the initial agent for hemodynamic support. Use of dopamine by peripheral vein has been included in guidelines, as resource constrain may preclude use of central lines.

There is enough evidence that early oxygen therapy, early aggressive fluid therapy to restore intravenous volume, and use of dopamine in fluid refractory shock have brought the mortality down. These interventions can be easily applied even in resource-limited circumstances even at primary and/or secondary level health facilities.

Early appropriate antibiotics, correction of hypoglycemia, hypocalcemia, and avoiding hyperglycemia are recommended. Randomized controlled trials are needed to establish choice of inotropic and vasopressor therapy for initial management, dose, and timing of use of corticosteroids, administration of blood and blood products, protective mechanical ventilation, glycemic control, techniques of renal replacement therapy.

Studies show that compliance with published guidelines tends to be inadequate. Further research evaluating individual components of guidelines and relative benefit of each of these interventions in resource-limited setting is needed, as also the benefit of adherence with guidelines and standardized set orders.
